# The Paradox of Isochrony in the Evolution of Human Rhythm

**DOI:** 10.3389/fpsyg.2017.01820

**Published:** 2017-11-06

**Authors:** Andrea Ravignani, Guy Madison

**Affiliations:** ^1^Language and Cognition Department, Max Planck Institute for Psycholinguistics, Nijmegen, Netherlands; ^2^Veterinary and Research Department, Sealcentre Pieterburen, Pieterburen, Netherlands; ^3^Artificial Intelligence Lab, Vrije Universiteit Brussel, Brussels, Belgium; ^4^Department of Psychology, Umeå University, Umeå, Sweden

**Keywords:** synchrony, prediction, interaction, coordination, turn-taking, evolution of music, evolution of speech

## Abstract

Isochrony is crucial to the rhythm of human music. Some neural, behavioral and anatomical traits underlying rhythm perception and production are shared with a broad range of species. These may either have a common evolutionary origin, or have evolved into similar traits under different evolutionary pressures. Other traits underlying rhythm are rare across species, only found in humans and few other animals. Isochrony, or stable periodicity, is common to most human music, but isochronous behaviors are also found in many species. It appears paradoxical that humans are particularly good at producing and perceiving isochronous patterns, although this ability does not conceivably confer any evolutionary advantage to modern humans. This article will attempt to solve this conundrum. To this end, we define the concept of isochrony from the present functional perspective of physiology, cognitive neuroscience, signal processing, and interactive behavior, and review available evidence on isochrony in the signals of humans and other animals. We then attempt to resolve the paradox of isochrony by expanding an evolutionary hypothesis about the function that isochronous behavior may have had in early hominids. Finally, we propose avenues for empirical research to examine this hypothesis and to understand the evolutionary origin of isochrony in general.

## Contents

This paper deals with isochronous temporal patterns. The emphasis is on the quantitative properties of isochronous patterns, and their perception and production in humans. The paper is organized in seven sections, namely:

(1) *What is isochrony?*, where we lay out crucial definitions and summarize basic relevant concepts;
(2) *The relevance of isochrony to human music and speech*, where we discuss how isochrony might partly underlie some behaviors in modern humans, such as music, speech and dance;
(3) *Mathematics, physics and signal processing*, where we discuss isochrony from the pure perspective of its physical and mathematical structure (as opposed, for instance, to its biological, behavioral or cognitive nature);
(4) *Physiology and neuroscience*, where we suggest how isochronous patterns have biological and psychological relevance for living organisms;
(5) *Comparative cognition: Non-human animals*, where we briefly summarize previous empirical attempts in finding, either directly or indirectly, isochronous behaviors in other species;
(6) *Isochrony in interaction*, where we move from isochronous behaviors in single individuals to group behaviors potentially involving isochrony;
(7) *Evolutionary hypotheses and future empirical work*, where we join all strands laid out in the previous six sections, and sketch an evolutionary account for the origin of isochrony in our species.

The aim of this paper is not to provide an exhaustive review of each of these areas. Rather, we attempt to establish a first connection between as many explanatory levels of isochrony as possible, across scientific disciplines and research traditions.

## What Is Isochrony?

**FIGURE 1:**
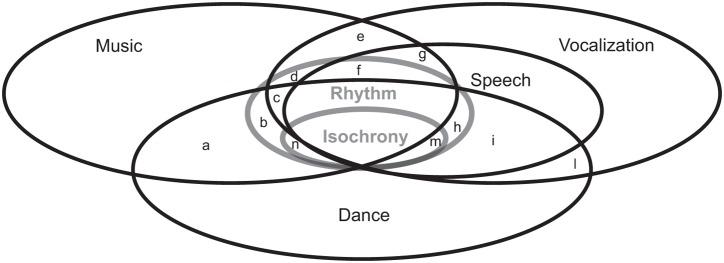
Conceptual relationships among music, vocalizations, speech, and dance with respect to temporal structure, rhythm, and isochrony. Lowercase letters a–n denote some possible intersections. For instance, *g* denotes shared timing between speech and music (but not dance), while *n* denotes isochronous music and dance with no commonalities with speech. It is up to empirical research to decide which ones of these are empty. Notice that the question of intersections is not moot: one of the first papers on the origins of dance (Laland et al., 2016), for instance, implicitly suggested that the evolutionary path for the emergence of dance had to go from *e* to *m* to *h*.

**FIGURE 2:**
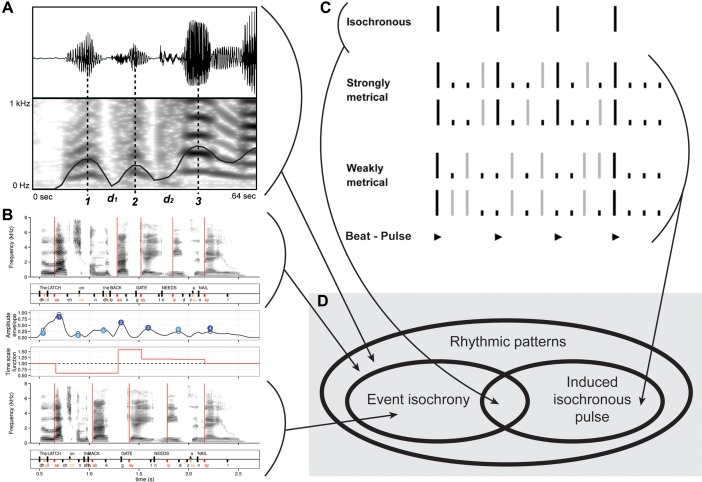
Rhythm, isochrony and pulse. **(A)** Examples of a non-isochronous speech rhythmic pattern. Figure modified from Jadoul et al. (2016). **(B)** Examples of another non-isochronous speech rhythmic pattern **(top)**, manipulated into isochrony **(bottom)**. Figure modified from Aubanel et al. (2016). **(C)** Musical rhythm showing event isochrony **(top)** and non-isochronous musical pattern generating isochronous expectations **(bottom)**. Figure modified from Launay et al. (2014). **(D)** Venn diagram showing the relationship among: rhythmic patterns vs. event isochrony vs. isochronous pulse perception.

Music is a complex phenomenon composed of interdependent parts. While a holistic approach is always important, the analytic, scientific method works by first analyzing constituent components individually (Fitch, 2015). Rhythm is a crucial dimension of human music. In common language, but also in scientific publications, different meanings are often conflated into the word ‘rhythm’ (see **Table 1** for definitions). In the most general definition, *rhythm* denotes a pattern of events in time (McAuley, 2010). An *isochronous* pattern is a rhythm where all intervals between events are equal, like those of a metronome. Hence, all isochronous sequences are rhythmic, but not vice-versa (**Figure 1**). A third related concept is the *beat*, namely the psychological tendency to superimpose an isochronous grid to a rhythmic sequence (a.k.a. pulse or beat perception, **Figure 2C**). The focus of the present paper is the evolutionary significance of the human perception and production of rhythmic sequences that are physically isochronous, henceforth simply ‘isochronous.’ We deliberately avoid discussing pulse and beat perception, as these have been object of much empirical research and many theoretical frameworks. Pure isochrony has received comparatively less attention.

**Table 1:** Definitions of key concepts discussed in the paper (Expanded and modified from Ravignani and Norton, 2017).

Name	Definition
Rhythm	Pattern of events in time; i.e., ‘a specific succession of durations’ (Wade, 2004, p. 57).
Interval	Temporal duration encompassed by two events.
Idealized isochrony	A rhythmic pattern where all intervals have *equal duration*.
Isochrony (empirical)	A rhythmic pattern where all intervals have *roughly* equal duration. Alternatively, a rhythmic pattern obtained by jittering events in an idealized isochronous pattern.
Anisocronous	Not isochronous. Operationally, a pattern exhibiting less isochrony than another pattern under consideration.
Pulse a.k.a. beat	Psychological tendency to superimpose an isochronous grid (not necessarily present in the physical signal) to a rhythmic sequence. In some cases, such as here, pulse and beat are defined as equivalent. In some other cases, the beat is defined as the isochronous grid generated via metrical expectations (see below).
Synchronous	Two rhythmic patterns where each event in one pattern occurs at the same time as a corresponding event in the other pattern.
Meter	Hierarchical organization of temporal events based on stress and other spectral properties, such as loudness alternation, pitch variation, etc.
Syllable-timed	Language where all accented and unaccented syllables are isochronous.
Stress-timed	Language where all stressed syllables occur isochronously.
Mora-timed	Language where all moras, consisting in syllables or some syllable combinations, are isochronous.
Endogenous	Pattern production involving a ‘self-sustained clock that governs the rhythm’ (Pikovsky et al., 2003, p. 87).
Exogenous	Pattern production that is influenced by an *external* oscillator/clock/pacemaker.
Reactive	Exogenous mechanism of production of a temporal interval whose duration is a direct *response* from a previous duration in the environment.
Predictive	Exogenous mechanism of production of a temporal interval whose duration is based on *expectations* about an upcoming environmental duration.
Entropy	The expected value of the information content of a signaling system. It quantifies the *amount of information* in a signal, without any reference to its meaning.
Redundancy	A measure of the actual information content relative to the maximum possible information content of a signaling system. High redundancy means that information is more ‘diluted.’
Compressibility	Here: inverse of entropy.
Expressivity	Capacity of a signaling system to convey different *meanings*.


Humans are particularly good at producing and perceiving both rhythmic and isochronous patterns (Bolton, 1894; Fitch, 2009, 2015; Motz et al., 2013; Ravignani et al., 2017). Yet, while general rhythm capacities could be biologically useful for foraging, mating, navigating the environment and predicting events, propensity for isochrony does not seem to confer any evolutionary advantage to modern humans (Fitch, 2009). Humans’ aptitude for isochrony contrasted with its apparent lack of evolutionary function constitutes the paradox of isochrony. Below we offer some perspectives on the presence of isochrony in nature and in modern humans’ everyday life.

Humans have extraordinary abilities to deal with isochronous behaviors. We can detect deviations from isochrony on the order of 20 ms or 4% of the interval to be timed, but we can also perceive the underlying isochrony even in the face of random deviations and gradual increases/decreases in intervals (Bolton, 1894; Madison and Merker, 2002, 2004; Max and Yudman, 2003; Repp, 2005; Madison, 2009; Motz et al., 2013; Repp and Su, 2013). When confronted with isochronous sequences where intervals have been slightly jittered, humans will tend to regularize the intervals and perceive the whole sequence as isochronous (for psychophysical thresholds see Friberg and Sundberg, 1995; Madison and Merker, 2002; Merker et al., 2009). Also when producing isochronous sequences the variability is around 4%, but it can be decreased to about 2% of the interval when synchronizing to a sound sequence with faster metrical levels (Madison, 2014). Listening to an isochronous sequence typically induces a beat (**Figure 2C**), which in turn generates expectations that future events will fall into a multiple or integer subdivision of the beat. A violation of the expectation, such as slightly changing the onset of one event, can be measured by mismatch negativity (Motz et al., 2013). The processes underlying this reaction are subliminal (Madison and Merker, 2004) and require no learning, which indicates that the beat is a very basic, inherited phenomenon.

In evolutionary biology, a behavioral trait can appear for a number of reasons. It can be an evolutionary *adaptation*, namely a trait which evolved to increase a species’ fitness in a given environment. As such, it may more or less have lost its adaptive value due to changes in the environment, while still prevailing in the population because it has not been selected against. It can also be a by-product of other evolutionary processes, a so-called *exaptation*. As such, isochrony might have been recruited for purposes unrelated to the pressures which caused its early emergence (Merker et al., 2009). In the present context, isochrony refers to humans’ perception and production of isochronous event sequences within the bounds and constraints reviewed above.

## The Relevance of Isochrony to Human Music and Speech

**FIGURE 3:**
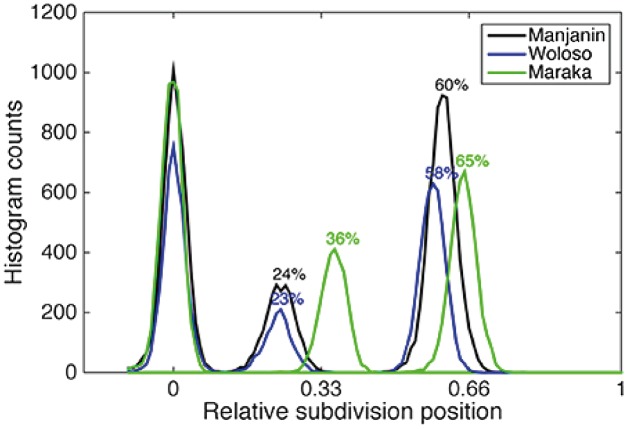
Complex metrical structure found in djembe drumming from Mali. In this musical culture, while the top level of temporal organization is isochronous, the level below it is neither isochronous nor exhibits small integer ratios. For three musical pieces (Manjanin, Woloso, and Maraka), the figure shows the time of occurrence of other beats between two isochronous beats. Manjanin and Woloso are clearly anisochronous, while Maraka (green plot) is almost isochronous. Figure copied verbatim from Polak et al. (2016).

The roots of the human propensity for isochrony are clearly found in our biology, specifically in some preparedness of our neural system (Buzsaki, 2006; Arnal and Giraud, 2012; Fujioka et al., 2012; Fujii and Wan, 2014; Merchant et al., 2015). For example, newborn babies react differently to isochronous than to anisochronous sequences (Honing et al., 2009). Similarly, children aged 2–4 years show motoric isochronous behavior with clear periodicities, though little tempo adjustment (Eerola et al., 2006). Although isochrony in music is a human universal (**Figure 2C**), there is considerable variation across the worlds’ music cultures. Western musical cultures appear to employ isochrony most thoroughgoingly, for example with rhythmic sequences being composed of isochronous subsequences (e.g., Bach). When rhythmic patterns are not isochronous, they are based on a psychologically induced sense of beat (Merker et al., 2009). In these cases, notes that are played continuously confirm or violate the induced pulse (**Figure 2D**), either in a structural or expressive manner (see Merker, 2014 for a novel perspective). African music is also isochronous at some descriptive level (though see **Figure 3**), while Asian music tends to be less so. Some North-American Indian, Javanese Gamelan, and Western electro-acoustic traditions exhibit no isochrony at all, but it might be argued that they do not fulfill reasonable definitions of music. For comparisons of timing in different musical cultures, see (Arom, 1991; Polak et al., 2016; Neuhoff et al., 2017). Although humans are cognitively biased toward isochrony in music (Ravignani et al., 2016a; Fitch, 2017), this bias is apparently modulated by enculturation (Jacoby and McDermott, 2017, though see Bowling et al., 2017). Finally, isochrony is often associated with motor synchronization in the literature. However, a recent medical case study has found a dissociation between perception of isochrony, among others, and audio-motor synchronization abilities (Bégel et al., 2017).

Speech is another human activity which may involve isochrony (**Figure 1**). The research field investigating rhythmic regularities in speech has been split for decades (Lehiste, 1977; Roach, 1982; Kotz and Schwartze, 2010, 2016; Fujii and Wan, 2014). Some scholars argue that world languages can be classified in groups exhibiting isochrony at the sentence, mora, or syllable levels (called, respectively, stress-timed, mora-timed, and syllable-timed languages, see **Table 1** and Grabe and Low, 2002; Fabb and Halle, 2012). Other researchers argue the opposite, namely that the speech signal is inherently anisochronous (**Figures 2A,B**), and the feeling of isochrony derives, for instance, from perceptual regularization rather than physical properties of the signal (Tuller and Fowler, 1980; Dauer, 1983; Jadoul et al., 2016; Brown et al., 2017). Without entering this debate here, some empirical findings are worth noticing. In particular, no matter the theoretical perspective adopted, human *vocalizations* can be experimentally driven toward isochrony (Jacoby and McDermott, 2017), especially when two individuals are asked to speak synchronously (Bowling et al., 2013) or perform turn-taking (Schultz et al., 2016). If speech recordings are experimentally manipulated, so that the syllable timing follows heterogeneous rhythmic patterns, isochronous speech is more intelligible than anisochronous speech (Aubanel et al., 2016). Finally, a recent experiment found evidence for isochronous timing in children’s handwriting (Pagliarini et al., 2017).

A third common human activity where isochrony is hypothesized to play a role is dance (Fitch, 2016; Laland et al., 2016; Richter and Ostovar, 2016; Su, 2016a,b). Similarly to music, a series of isochronous events, such as a drum line, may provide anchor points in time used to structure dance movements (Fitch, 2016; Laland et al., 2016). Likewise, biophysical constraints on movement produce isochronous or integer ratio temporal intervals (e.g., Merker et al., 2009; Su, 2016a). This isochrony-centered perspective might however be quite specific for dance in humans inhabiting the Western world. A more inclusive approach considers dance a polyhedric behavior present in all human cultures, and whose precursors can be found in other animal species (Fink and Shackelford, 2016; Ravignani and Cook, 2016). If this approach is adopted, then isochrony might not be such an indispensable pillar of dance (Ravignani and Cook, 2016).

All the above suggests that, while isochrony might not be crucial in dance or speech, it is present in human everyday musical behavior (**Figure 1**). So, why is isochrony so common if it doesn’t appear to serve any particular function (Fitch, 2009; Merker et al., 2009), at least in modern humans? Below we will try to analytically decompose isochrony even further in its constituent parts across disciplines.

## Mathematics, Physics, and Signal Processing

**FIGURE 4:**
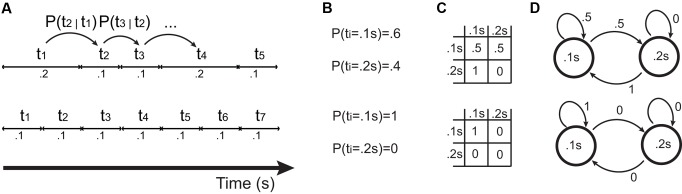
Different ways of representing patterns of time intervals. The bottom shows an isochronous pattern, and the top a slightly more complex pattern. **(A)** Time series of intervals (t_1_, t_2_, …), inducing transition probabilities P(t_1_ = x | t_2_ = y), shortened as P(t_1_ | t_2_). **(B)** Set of interval durations and probabilities of occurrence. **(C)** Transition matrices resulting from transition probabilities. **(D)** Equivalently, a probabilistic finite state machine also generates the patterns shown in **(A)** and described by the transition matrix in **(C)**.

Rhythms, including isochronous ones, can be formalized mathematically (Cohen, 1962; Toussaint, 2013). From a purely information-theoretic perspective (MacKay, 2003), when producing a signal over time, isochrony minimizes the signal’s entropy. A pattern of time intervals (**Figure 4A**) can be described by a set of interval durations and the probability of occurrence of each interval (**Figure 4B**). A more refined model features conditional transition probabilities (**Figures 4C,D**), where the duration of the upcoming interval is determined probabilistically from the duration of the previous *n* intervals (Cohen, 1962). An example could be a specific pattern for which, given that the first and second intervals are short, there is a high probability that the third interval is long. This is not the case for isochronous sequences: no matter which interval is to be predicted, and how many past intervals are taken into account, the upcoming interval will be a constant value equal to past intervals with probability 1. This property makes isochronous sequences, from an information-theoretic perspective, purely deterministic and predictable, granting the lowest possible entropy. In other words, conditional on a known repetition rate (i.e., tempo), isochrony minimizes entropy in rhythmic sequences.

Physics offers prime examples of isochronous processes (Strogatz, 2000). For instance, atomic clocks are based on isochronous oscillations, i.e., atomic activity regularly occurring at known frequencies (Strogatz, 2003). Events like these, reliably repeating at regular time intervals, can serve as a benchmark (**Table 1**): they are used by mankind to synchronize their clocks and, for our purposes, they represent the highest level of isochrony achievable by a system. In other words, empirical isochrony can be defined as synchrony, i.e., perfect co-occurrence (Ravignani, 2017a), of an empirical sequence with respect to an isochronous reference grid.

**FIGURE 5:**
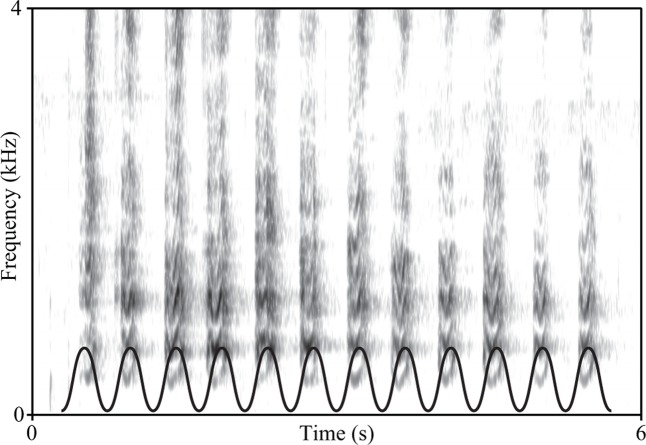
Spectrogram of a California sea lion (*Zalophus californianus*) bark sequence, showing an extremely isochronous rhythm (Fourier window length = 0.02 s). To highlight this metronomic regularity, we have superimposed a sinusoidal wave to the lower part of the spectrogram. The sinusoid was plotted to feature as many maxima as the number of barks: the fact that each maximum aligns with one bark provides an intuitive qualification of the isochronicity of this pinniped vocalization. (Recording collected by AR with a Zoom H6 recorder and Zoom XY-6 microphone at 44.1 kHZ, 16-bit, at Santa Cruz pier, California, on October 2016).

Likewise, mathematical and computational models of isochrony are quite straightforward. In the simplest case, isochrony can be mathematically generated by trigonometric functions, such as sine and cosine (**Figure 5**). More realistic models of isochronous human behavior involve, for instance, long-range correlations (Madison and Delignieres, 2009), and fractal scaling (Madison, 2004). Isochronous synchrony between two or more entities can be modeled using phase resetting (Sismondo, 1990; Greenfield and Roizen, 1993), period bisection (Ravignani, 2014; Ravignani et al., 2014b), coupled oscillator models (Strogatz and Stewart, 1993; Large and Kolen, 1994; Strogatz, 2000; Rouse et al., 2016; Ravignani, 2017a), and a number of other techniques (reviewed in Ravignani and Norton, 2017). In dynamical systems, isochrony ranks amongst the best understood non-linear processes. The take-home message is that isochrony, in many of its forms and variants, can be comprehensively defined by very simple mathematical expressions (and visualized geometrically, see Toussaint, 2013; Ravignani, 2017b).

## Physiology and Neuroscience

**FIGURE 6:**
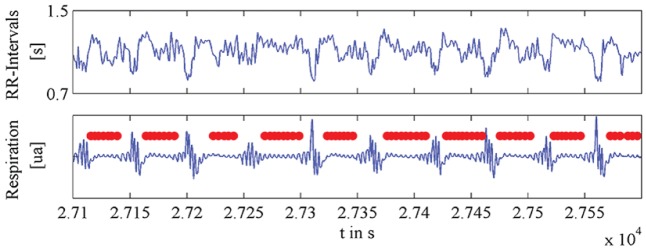
Plot of exemplary time series of human heartbeat **(top)**, and respiration **(bottom)**, during and after apnea events. Figure modified from Penzel et al. (2016).

Human heartbeat, respiration, and locomotion all have an element of isochrony (**Figure 6**) in that they exhibit more regularity than random patterns but less regularity than periodic patterns in physical systems (Winfree, 1986). On the one hand, these processes can be quite regular within a short enough window of measurement (Larsson, 2012, 2014, 2015; Teie, 2016). In fact, the most commonly used measures of these physiological variables (beats, breaths or gaits per minute) assume *local isochrony* for the sequence analyzed. So, for example, most finger tapping studies have collected sequences of only 20–50 intervals in order to avoid the complicating drift (Wing and Kristofferson, 1973a,b; Madison, 2001). On the other hand, heartbeat, respiration and locomotion are highly dynamic and mutable in order to be functional (**Figure 6**); in other words, acceleration, deceleration, and phase shifts – all disrupting perfect isochrony – are quite common (Strogatz, 2003).

Isochrony and synchrony are also emergent properties of the nervous system. Synchronous groups of neurons, each oscillating isochronously, are common in the brain (Buzsaki, 2006). At a higher level, cortico-subcortical networks are usually recruited to produce and perceive external isochronous events (Kotz and Schwartze, 2010, 2016; Nozaradan et al., 2011; Fujioka et al., 2012). Finally, some pathological states of the central nervous system are known to disrupt intentional isochrony, for instance Parkinson’s disease (Grahn, 2012).

What is the neurophysiological basis for behavioral isochrony? Interesting connections between timing of vocalizations and neurophysiology have been discovered by physiologists working on non-human animals, for instance, amphibians. In some frog species, the temporal structure of courtship vocalizations is modulated by hormones (Zornik and Kelley, 2011). An outstanding question is, of course, whether these connections between temporal behavior and hormones can be found in humans, whose ethogram might be more complex than some amphibians. Recent findings in the human neurogenetics of music make this line of research quite promising (Granot et al., 2007; Ukkola et al., 2009; Ukkola-Vuoti et al., 2013; Kanduri et al., 2015a,b).

## Comparative Cognition: Non-Human Animals

Some neural, behavioral and anatomical traits underlying isochronous rhythm perception and production are shared with a broad range of species (Wilson and Cook, 2016). These may either have a common evolutionary origin, or have evolved into similar traits under different evolutionary pressures (Ravignani et al., 2014b, 2016b). For instance, timing processes involving the basal ganglia and isochronous oscillations in the brain are shared with other primates and probably other animal taxa. Other traits are rare across species, only found in humans and a few other animals (Patel et al., 2009a,b). For instance, motor entrainment to an external isochronous pulse is only found sparingly in the animal kingdom (Fitch, 2015; Iversen, 2016; Ravignani et al., 2016b; Wilson and Cook, 2016).

A first, crucial difference separates human isochronous behavior from the examples of isochrony in nature provided above. This is the extent to which isochronous pattern production is driven and affected by external factors. A decaying isotope and a person walking at regular pace do not need an external oscillatory stimulus to keep producing isochronous behavior. These are cases of *endogenous* isochrony (Pikovsky et al., 2003), corresponding to self-sustained oscillators in physics. Conversely, the isochronous behavior exhibited by humans dancing or tapping to music is mostly *exogenous*: an internal pacemaker is partially corrected by externally perceived oscillatory activity (Merker et al., 2009), corresponding to forced or coupled oscillators in physics.

How about all shades of gray between these two extreme cases? Those animal species exhibiting isochrony are sparsely and heterogeneously divided among cases of endogenous and exogenous isochrony. This makes animal research key to understand the nature of human isochrony: for every particular type of isochrony found in a species, its neural mechanisms and resulting behaviors can be mapped and compared between that species and humans (Merchant et al., 2015).

Within exogenous isochrony, another distinction is between prediction and reaction (**Table 1**), depending on whether a timing event is produced by predicting when the next event should occur, or reacting to a previous event (Patel et al., 2009b). Humans exhibit exogenous *predictive* timing (Fujioka et al., 2012), while crickets exhibit exogenous *reactive* timing (Greenfield and Roizen, 1993). Finally, the isochronous tail wagging of dogs is quite likely to be endogenous, as external oscillatory stimuli are unlikely to affect its period or phase (cf. Buxton and Goodman, 1967; Fitch, 2009). While some species can be readily classified along the dimensions above (Rouse et al., 2016), for other species some data is available (**Figure 5**), though still not enough to be classified into isochrony types (e.g., Schusterman, 1977). Finally, for the majority of species, no systematic investigation of isochronous behavior has been performed. In other words, we still lack data on how most species produce and perceive isochrony under a wide range of different conditions, which would be diagnostic to the underlying mechanisms and limitations.

In animal research, isochrony has been investigated using two main methods: observing natural behavior and training animals to produce specific temporal sequences. Isochrony as natural behavior in other animal species has long been studied, though its relevance to human rhythm has been pointed out only recently (Madison, 2004; Merker et al., 2009; Ravignani et al., 2014b). In fact, many animals signal over time in a precisely isochronous fashion (e.g., see **Figure 5**). ‘Isochronous species’ span crickets, frogs, fireflies, birds, crabs, and marine mammals (Schusterman, 1977; Sismondo, 1990; Greenfield and Roizen, 1993; Strogatz, 2003; Merker et al., 2009; Kahn et al., 2014; Norton and Scharff, 2016). As these studies are often purely behavioral and observational, rarely targeting neurobiological brain mechanisms, it is difficult to know whether isochrony is endogenous, exogenous, predictive or reactive.

An alternative is to test animals’ capacities to produce isochrony in a controlled experimental setup. This is often done in conjunction with synchronization experiments. The animals are trained to produce specific isochronous behaviors, often with the purpose of entraining to a musical beat, and are then tested in their ability to generalize to different tempi and levels of jitter (Wilson and Cook, 2016). The only irrefutable results of *exogenous predictive isochrony* in any animal species are three: humans, a sea lion and a cockatoo (Patel et al., 2009a; Cook et al., 2013). Trained isochrony due instead to *reactive* timing might be more common: several species appear capable of producing series of temporal intervals of equal duration (Hasegawa et al., 2011; Hattori et al., 2015).

## Isochrony in Interaction

In human communication, two opposing functions affect the structure of the signal: expressivity and compressibility (**Table 1** and Kirby et al., 2015). Expressivity influences the amount of information content, hence semantics, which ideally should be maximized (Kirby et al., 2015). Compressibility refers to the density of information transmitted: intuitively, it is cost efficient to transmit the same amount of information in its shortest or maximally compressed form (MacKay, 2003). In other words, signalers would ideally broadcast the maximum quantity of information, using the least possible amount of signal. This tradeoff is further modulated by redundancy: a maximally compressible communication system with no redundancy can be irreversibly corrupted by a minimal transmission error. Hence, a signaler might not want to completely minimize entropy in order to leave room for redundancy.

**FIGURE 7:**
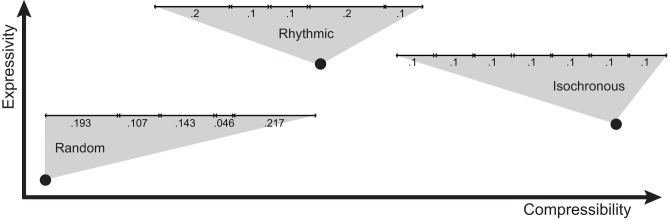
Compressibility-expressivity tradeoff for temporal patterns. Three examples of timing patterns are provided, placing them in this compressibility-expressivity space. For each example, the black dot denotes the approximate location of the pattern. Each horizontal line represents one pattern, with the duration of each chunk expressed in seconds below it. Patterns used for communicative purposes are predicted to lie on or near the main diagonal, and patterns in the top-left and bottom-right quadrants of the diagram are hence not predicted to serve communicative purposes.

To what extent can compressibility, expressivity, and redundancy account for human isochrony (**Figure 7**)? Mathematically, isochronous signals maximize redundancy and minimize entropy, but leave almost no room for expressivity. Comparatively, when human participants develop signaling systems in communication experiments, no expressivity leads to maximum compressibility (Kirby et al., 2008, 2015). This, by analogy, would dismiss isochronous pattern production as an expressive communication system (**Figure 7**), i.e., a system where signals are mapped to meanings (but see Bharucha and Pryor, 1986; Horr and Di Luca, 2015). However, the meaning of the transmitted message could lie in the signal emission *per se*, rather than that being broadcasted through the signal. This is the concept behind ‘signaling signalhood’ (Scott-Phillips et al., 2009): the message of an isochronous pattern is its ‘isochronicity,’ instead of being used in referential communication. Consider a hypothetical example. Take rhythmic sequences composed of only two durational intervals (such as the first two intervals of a sequence in **Figure 4A**). The set of all two sequences could be used for communicative purposes in two main ways. In the first, more common case of ‘referential communication,’ the duration of the two intervals could encode different conceptual properties. For instance, the first interval could be used to encode the size of a referent, while the second its brightness. Hence, a rhythm composed of a short and a long interval would communicate a small, dark object, while a long and a short interval would refer to a large bright object. This variability in the lengths of the intervals would grant expressivity. However, if isochronous sequences were the signals most frequently transmitted, this way of encoding signals could not be expressive, because all objects would end up being encoded as having an average size and brightness. In contrast, in the second case of ‘signaling signalhood,’ the two intervals would be used to communicate precision in isochronous pattern production, i.e., to signal isochronicity. Hence, a pattern as the bottom of **Figure 4A** would signal high precision in isochrony, while the top pattern in **Figure 4A** would signal poor isochrony. From this point of view, human perception and production of isochronous patterns might better fit the second, signaling signalhood framework, rather than the first, referential communication framework.

There is a close match between the most precise levels of isochrony that humans are capable of producing and those they are capable of perceiving (Madison and Merker, 2002; Merker et al., 2009). This match also offers some support for the hypothesis that isochrony might have been shaped for communicative purposes. In other words, a communication system, and in particular one that takes advantage of, and evolves from, perceptual biases (Ryan, 1998), will show a match between features of the signal and the capacities to perceive those features. For example, the plumages of many bird species reflect ultraviolet light, which humans and other species cannot see, while conspecific birds can readily perceive and use to select a mate (Andersson and Amundsen, 1997; Vorobyev et al., 1998; Eaton, 2005). We hypothesize that an analogous process might have resulted from isochrony (expanding on Merker, 1999, 2000), if this were a communicative trait. In particular, a communication system employed to transmit information about deviations from an isochronous pulse would evolve toward levels of precision comparable between production and perception (Merker, 1999, 2000). This comparable precision is exactly what can be observed in human motoric and perceptual isochrony (Madison and Merker, 2002; Merker et al., 2009), offering some preliminary, indirect support for a possible communicative function of isochrony.

**FIGURE 8:**
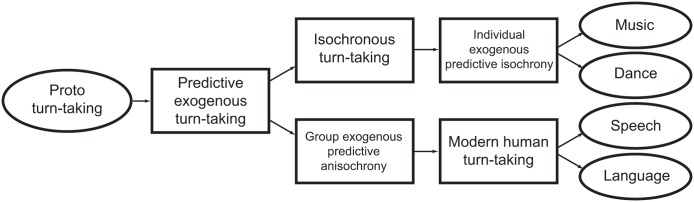
Flux diagram of hypothetical precursors to isochronous synchrony and turn-taking. A hypothetical form of ‘proto turn-taking’ branches in two, leading to isochrony, as found in music and dance, and anisochronous turn-taking, as found in language and speech.

**FIGURE 9:**
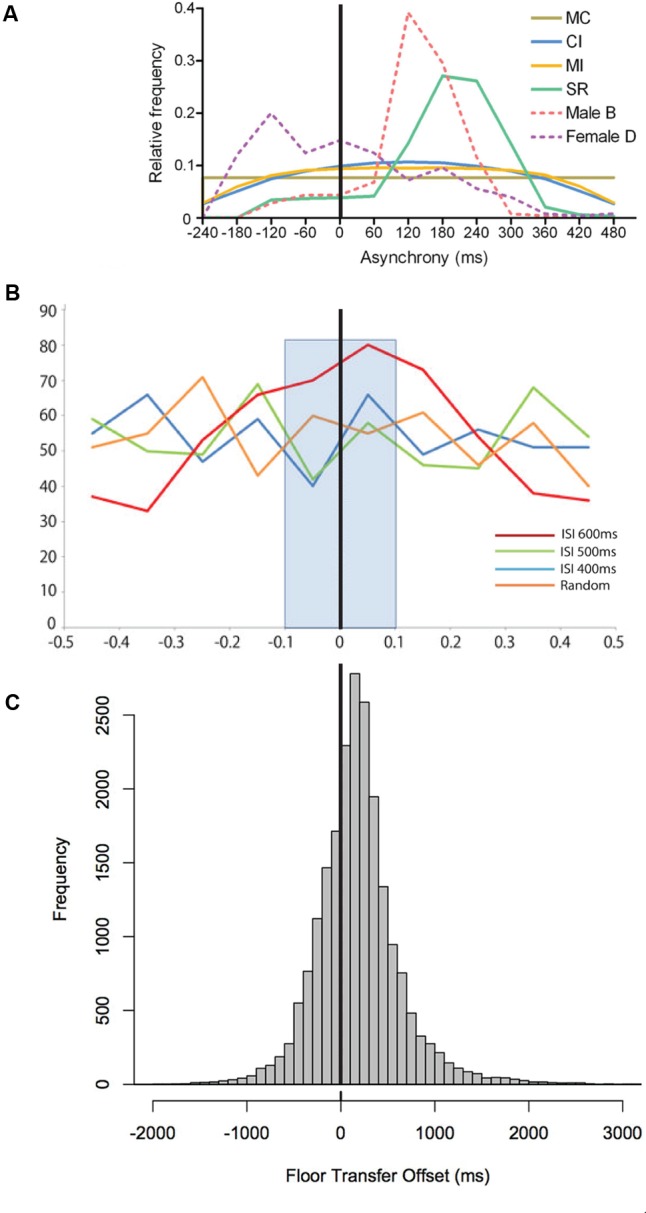
Onset asynchronies in **(A)** a synchronization experiment in budgerigars (each bird, Male B and Female D, is a dashed line), **(B)** a tapping experiment in one individual chimpanzee, and **(C)** turn-taking in human conversation. Figure panels modified from Hasegawa et al. (2011) for **(A)**, Hattori et al. (2013) for **(B)**, and Levinson and Torreira (2015) for **(C)**.

Isochrony does not appear to be used in the overt communication of modern humans, but might have played a role in some form of communication employed by our ancestors. In fact, isochrony is the optimal way to establish synchronized group signaling because it makes the duration of next interval perfectly predictable by another person or conspecific (Merker et al., 2009). This musical perspective on the evolution of isochrony connects to turn-taking, which is a crucial component of human language (**Figure 8**). Turn-taking allows speakers to effectively interact in conversation: it avoids that speakers’ utterances overlap, while still enabling utterances to occur within a reasonable amount of time from each other. Interestingly, turn-taking in language is both predictive and exogenous, but seems to lack isochrony, except maybe in a few special cases. Still, turn-taking exhibits a particular temporal structure (Stivers et al., 2009; Levinson and Torreira, 2015). This structure appears to arise by a constant 200 ms lag (**Figure 9C**) between the ends and starts of utterances across cultures (Stivers et al., 2009), rather than a lag between the starts of consecutive utterances. This fixed-interval delay contrasts with the slightly positive or negative lags found in animal synchronization experiments (**Figures 9A,B**), and the anticipatory reaction in human musical synchronization. So, in modern humans, turn-taking is far from isochrony (except for when it is a by product of utterances having the same duration within and between speakers), but it might promote isochrony (Schultz et al., 2016). This makes turn-taking in modern organisms a potential approach to understand the evolution of isochrony (see **Figure 8**).

## Evolutionary Hypotheses and Future Empirical Work

In conclusion, isochrony does not appear to relate to any *current* selection pressure. This is not surprising: a large number of evolved, heritable traits do not readily map to clear selection pressures in extant species. Instead, the pressures giving rise to isochrony might be sought in ancient humans, operating at some point between now and the split between our ancestor and that of chimpanzees/bonobos. The most articulate hypothesis to date proposes a multistage model (Merker, 1999, 2000; Merker et al., 2009). According to this, a recent ancestor to modern *Homo sapiens* would have been exposed to a selection pressure to attract migrating conspecific females. Accordingly, group vocalizations would have provided a conspicuously loud signal. The more individuals that managed to synchronize their calls, the greater the sound intensity, and the farther its reach in the terrain. In turn, the easiest way to achieve synchronization is to produce an isochronous signal, which is maximally predictable by the other callers, leading to an isochronous, synchronous chorus (Merker et al., 2009). We further extend this idea, suggesting that early stages of vocal coordination could be precursors to two modern human traits: isochronous signaling in music and anisochronous turn-taking in language (**Figure 8**).

Our perspective focuses on the *function* of isochrony (Merker et al., 2009; Ravignani et al., 2014b), rather than its underlying ontogeny and phylogeny (Tinbergen, 1963). Hence, most ideas presented here are in principle compatible with other hypotheses focused on developmental trajectories or mechanisms (Iversen, 2016; Teie, 2016). The functional reason why isochrony appeared in human evolution might therefore not be directly derivable from how isochrony appears in development, or from its neurobehavioral mechanisms. The mechanisms underlying isochrony which manifest through development (Eerola et al., 2006) might also be reflected in evolution, though they do not need to be (see Gould, 1977, and subsequent debate).

Research on modern humans provides some grounds, in principle, for isochrony to have been an evolutionary selected trait. In particular, isochronous timing seems quite variable across individuals, and enhanced by learning (Max and Yudman, 2003; Manning and Schutz, 2016; Tierney et al., 2017). Individual differences and learning plasticity are neither necessary nor sufficient conditions to show that a trait, such as isochrony, underwent evolution by natural selection. However, individual differences are often a prerequisite for natural selection to act on a trait. Likewise, learning plasticity can be an outcome of an evolutionary process acting on behavior and cognition, rather than on a physical trait.

For the purpose of the present paper, it would be interesting to find the genetic and neuro-hormonal biological substrates responsible for perception and production of isochronous behavior both in humans and other animals. In fact, as isochrony appears as a relatively simple behavioral trait, study of its neuro-genetic and hormonal substrates might prove an initial building block to understand rhythm more in general.

Our suggestions can be readily tested along several strands of empirical research in humans. Temporal interactions in groups of animals are known to lead to isochrony as one of the equilibrium outcomes (Sismondo, 1990; Greenfield and Roizen, 1993; Kahn et al., 2014). Human data on turn-taking may be re-analyzed, asking whether the isochrony of each partner entails the best predictability of turn-taking vs. constant-lag alternation. Likewise, the large body of research on isochrony perception and production across modalities and domains may be synthetized (Iversen et al., 2015; Celma-Miralles et al., 2016), to examine (1) the limits and boundaries of the human sense of isochrony, and (2) which experiments are lacking that would entail comparability across domains and modalities. In general, the field of rhythm would benefit by a tighter connection between individual and group processes: individual behavioral traits do not evolve in a vacuum, and individual timing might be modulated by social factors (Ravignani et al., 2014a,b; Ravignani and Cook, 2016; Schirmer et al., 2016). For instance, in some primate and avian species, singing is accurately timed with the group depending on the sex and social status of each individual singer (Mann et al., 2006; Gamba et al., 2016). With this logic in mind, we can list a number of specific outstanding questions:

• Do individual and group isochrony influence each other (Ravignani et al., 2014b)? How are they related at a mechanistic, functional, developmental and phylogenetic level (Kirschner and Tomasello, 2009; Ravignani et al., 2016b)?
• What are the effects of training, conformity, and social cues on the perception and production of isochronous behaviors (Schirmer et al., 2016)?
• How do different individual group interaction modes, for instance coordination and competition, map on to temporal patterns produced, such as isochrony (Ravignani et al., 2014a)?

These questions are not only relevant from the perspective of human cognitive neuroscience and animal behavior. They are also key to test evolutionary hypotheses, where the fitness landscape might be influenced by social factors and cultural niches (Tomasello, 2009; Boyd et al., 2011; Kendal, 2011).

Finally, while selection pressures in our ancestors are difficult to reconstruct, their effects might still be observable in the behavioral tendencies, genome and neuroendocrine system of modern humans (Holmquist and Vestin, 2010; Madison, 2011; Björk, 2013; Madison et al., 2017, in press). For instance, recent studies have mapped musical and rhythmic phenotypes to genes and hormonal profiles (Mosing et al., 2015; Miani, 2016a,b); more focused studies linking biology and psychology are needed for the specific trait(s) underlying isochrony.

## Author Contributions

AR wrote a first draft of the manuscript. GM provided references, guidance, and advice, and edited the manuscript.

## Conflict of Interest Statement

The authors declare that the research was conducted in the absence of any commercial or financial relationships that could be construed as a potential conflict of interest.
